# Building a pseudo-atomic model of the anaphase-promoting complex

**DOI:** 10.1107/S0907444913018593

**Published:** 2013-10-12

**Authors:** Kiran Kulkarni, Ziguo Zhang, Leifu Chang, Jing Yang, Paula C. A. da Fonseca, David Barford

**Affiliations:** a Division of Structural Biology, Institute of Cancer Research, Chester Beatty Laboratories, 237 Fulham Road, London SW3 6JB, England

**Keywords:** anaphase-promoting complex, single-particle electron microscopy, pseudo-atomic model

## Abstract

The anaphase-promoting complex (APC/C) is a large E3 ubiquitin ligase that regulates progression through specific stages of the cell cycle by coordinating the ubiquitin-dependent degradation of cell-cycle regulatory proteins. Depending on the species, the active form of the APC/C consists of 14–15 different proteins that assemble into a 20-­subunit complex with a mass of approximately 1.3 MDa. A hybrid approach of single-particle electron microscopy and protein crystallography of individual APC/C subunits has been applied to generate pseudo-atomic models of various functional states of the complex. Three approaches for assigning regions of the EM-derived APC/C density map to specific APC/C subunits are described. This information was used to dock atomic models of APC/C subunits, determined either by protein crystallography or homology modelling, to specific regions of the APC/C EM map, allowing the generation of a pseudo-atomic model corresponding to 80% of the entire complex.

## Abstract 2

This article describes an example of molecular replacement in which atomic models are used to interpret electron-density maps determined using single-particle electron-microscopy data.

In this article, we describe an example of molecular replacement in which we use atomic models to interpret electron-density maps determined using single-particle electron-microscopy data. The method of combining high-resolution structural information from crystallography with lower resolution structures obtained through electron microscopy is referred to as the hybrid method. We will briefly explain how the APC/C functions to regulate the cell cycle before describing our approach to determining a pseudo-atomic structure of the complex.

The APC/C is a large multi-subunit complex that functions as an E3 ubiquitin ligase to regulate progression through the cell cycle by mediating the destruction of cell-cycle proteins *via* the ubiquitin proteasome pathway (Peters, 2006 ▶; Sullivan & Morgan, 2007 ▶; Barford, 2011 ▶; Pines, 2011 ▶). The APC/C controls the destruction of proteins such as securin, cyclins and the Aurora and Polo mitotic kinases, the activities of which inhibit progression through distinct cell-cycle phases. The destruction of securin at metaphase triggers sister chromatid segregation and the onset of anaphase at mitosis. The APC/C interacts with its substrates through destruction motifs, predominantly the D box and KEN box, mediated by co­activator subunits that bind to the core APC/C during mitosis and G1.

The complex functions of the APC/C are reflected in its complex organization and size. Human APC/C is an assembly of 15 different proteins including the coactivator subunits (Table 1 ▶). Their masses range from around 200 kDa for Apc1 to 9 kDa for the smallest subunit Cdc26. We know from native mass spectrometry and crystallography that many subunits are present as two copies per complex, so that the overall mass is around 1.3 MDa, depending on the species. Knowing the subunit stoichiometry of a complex is important for accurate fitting of atomic models into the EM structure. *Saccharomyces cerevisiae* and *Schizosaccharomyces pombe* APC/C differ from metazoan APC/C by lacking the tetratricopeptide (TPR) subunit Apc7.

One striking feature of the APC/C is that only four proteins are involved in directly recognizing target proteins and in catalyzing the assembly of a polyubiquitin chain onto the substrate. All other subunits, which account for 80% of the mass of the APC/C, provide scaffolding functions to organize the catalytic and substrate-recognition subunits. Another feature of the APC/C is that many of the scaffolding proteins are structurally related. In human APC/C there are four TPR subunits that have very simple architectures based on multiple contiguous copies of a 34-­amino-acid sequence motif called the tetratricopeptide (TPR) motif. The smaller TPR accessory subunits stabilize the larger TPR subunits and also mediate inter-TPR interactions. In isolation, these TPR accessory subunits are disordered and they only assume a defined conformation when associated with the TPR subunits.

Our research has been aimed at obtaining atomic structures of individual APC/C subunits and to define how these subunits are organized within the whole complex as a means to understand how the APC/C recognizes its substrates and catalyses the assembly of polyubiquitin chains. To apply the hybrid approach to generate pseudo-atomic structures of the APC/C we have determined atomic models of most of the large APC/C subunits through a combination of protein crystallography and homology modelling. We now have atomic models for most of the large APC/C subunits except for Apc4 and the N-terminus of Apc1. We also lack structural information on some of the smaller APC/C subunits (Table 1 ▶).

An example of one of the canonical TPR subunits, Cdc16 associated with its accessory subunit Cdc26, is shown in Fig. 1 ▶(*a*) (Zhang, Kulkarni *et al.*, 2010 ▶). Two Cdc16 molecules self-­associate to form V-shaped homodimers. Each Cdc16 subunit consists of 14 contiguous TPR motifs that create an array of 28 antiparallel α-helices generating a right-handed TPR superhelix with two complete turns of superhelix. The other TPR subunits (Cdc23 and Cdc27) have similar architectures to Cdc16 (Fig. 1 ▶ *b*), as does Apc7 (not shown; Zhang, Roe *et al.*, 2010 ▶; Zhang *et al.*, 2013 ▶). The crystal structure of Apc10, a subunit that functions as the D-­box co-receptor in cooperation with an activator subunit (either Cdc20 or Cdh1), has been determined (Au *et al.*, 2002; Wendt *et al.*, 2001 ▶; Fig. 1 ▶ *c*). We have derived homology models for some APC/C subunits that we have not yet crystallized. The APC/C catalytic centre, formed by the cullin subunit Apc2 associated with the RING-domain subunit Apc11, was modelled based on the SCF structure (Fig. 1 ▶ *d*; Zheng *et al.*, 2002 ▶). The PC (proteasome–cyclosome) domain of Apc1 was modelled on the PC domain of Rpn2, a proteosomal subunit, the structure of which we recently determined (Fig. 1 ▶ *e*; He *et al.*, 2012 ▶). Interestingly, in addition to the TPR subunits, the PC domain of Apc1, and the coactivator subunits, are based on multiple repeat motifs. Coactivators are seven-bladed β-­propeller proteins as shown for the structures of Cdc20 (Fig. 1 ▶ *f*) and Cdh1 (Chao *et al.*, 2012 ▶; He *et al.*, 2013 ▶).

We used single-particle electron microscopy to determine three-dimensional reconstructions of the whole APC/C. Two views of a three-dimensional reconstruction of the *S. cerevisiae* APC/C based on cryo-EM data calculated to 11 Å resolution are shown in Fig. 2 ▶ (da Fonseca *et al.*, 2011 ▶). Overall, the APC/C adopts a triangular shape measuring 250 Å in the longest dimension. The molecule has an open lattice-like appearance and there are clear indications of rod-like and curved tubular densities, some of which correspond to the TPR superhelices and N-terminal cullin repeats of Apc2. To interpret this EM structure, and to generate a pseudo-atomic model of the APC/C, we have to locate the position of atomic models of APC/C subunits within the molecular envelope. We applied three approaches to assign regions of the electron-density map to specific subunits or domains. One approach to segment the APC/C EM density is to generate subcomplexes of the APC/C missing defined subunits. By determining the three-dimensional structures of these subcomplexes and comparing them with the holo APC/C, the difference densities can be assigned to the missing subunits (da Fonseca *et al.*, 2011 ▶; Schreiber *et al.*, 2011 ▶).

To generate the holo APC/C and define subcomplexes, we overexpressed recombinant APC/C using the MultiBac insect-cell expression system (Schreiber *et al.*, 2011 ▶). To reconstitute the budding-yeast APC/C of 13 proteins, we generated two baculoviruses for co-infection in insect cells. This system works well and we can purify 2–3 mg of APC/C per 5 l of insect cells, an increase in yield of some 500-fold compared with endogenous APC/C (Schreiber *et al.*, 2011 ▶). We observe a direct correspondence between endogenous and recombinant APC/C when the APC/C is assessed using silver-stained SDS–PAGE. The recombinant APC/C is active as an E3 ubiquitin ligase dependent on the coactivator, and D-box and KEN-box destruction motifs within cyclin. Thus, recombinant APC/C recapitulates the activity of endogenous APC/C.

We determined three-dimensional reconstructions of the recombinant holo APC/C and a variety of subcomplexes using negative-stain electron microscopy. For example, the sub­complex TPR6 comprises the TPR subunits Cdc16, Cdc23 and Cdc27 together with the TPR accessory subunits, whereas the remaining subunits, including the substrate-recognition and catalytic subunits, assemble into a subcomplex termed SC8 that is dependent on Cdc23 for its stable assembly. Both TPR6 and SC8 match their corresponding densities within the holo APC/C, showing that they adopt stable autonomous structures.

The first approach to dock atomic models of APC/C subunits into the APC/C EM structure was to define difference densities by selective subunit deletions (da Fonseca *et al.*, 2011 ▶; Schreiber *et al.*, 2011 ▶). In this method, we compare the structures of pairs of APC/C subcomplexes that differ in their composition by a specific subunit. This is illustrated in Fig. 3 ▶(*a*) for Cdc16, in which we compare two APC/C sub­complexes, of which only one incorporates Cdc16. The Cdc16 difference density defines both the position of Cdc16 within the molecular envelope and also the overall shape and molecular boundaries of Cdc16. The difference density has an elongated V-­shaped structure with twofold symmetry that exactly matches the atomic model of the Cdc16 homodimer (Zhang, Kulkarni *et al.*, 2010 ▶). We could apply a similar approach to Cdc27 by comparing the holo APC/C with APC/C lacking the Cdc27 subunit. The Cdc27 difference density, at the top of the TPR lobe, has a twofold-symmetrical structure that matches the twofold symmetry of the Cdc27 homo-dimer atomic model (Fig. 3 ▶ *b*).

We applied the subunit-deletion approach to five APC/C subunits (Table 1 ▶). A disadvantage of the method is that it is restricted to cases in which the resultant subcomplexes are large enough and stable enough to be analysed by negative-stain electron microscopy. Some subunits play critical scaffolding functions and their deletion prevents the assembly of usably sized subcomplexes. For example, deletion of Cdc23 results in a failure to generate APC/C subcomplexes of reasonable size. However, we could define the position and molecular boundaries of Cdc23 by superimposing the subcomplexes TPR6 and SC8 onto the intact holo APC/C structure. TPR6 and SC8 share Cdc23 in common, and therefore their overlapping densities can be assigned to Cdc23. The Cdc23-assigned density (Fig. 3 ▶ *c*) has an almost identical structure to that assigned for Cdc27, related by an approximate twofold rotation about Cdc16. This is consistent with Cdc23 and Cdc27 being paralogues.

The second approach to map atomic models is to dock directly into EM maps by visual inspection. This method is possible when regions of the EM maps display strong structural features characteristic of the atomic model. This procedure becomes more reliable when the majority of the EM map has been assigned through empirical approaches and the docking can be guided by additional data such as antibody labelling or protein–protein interaction data. For example, the cullin subunit Apc2 has a rod-like N-terminus and a globular C-terminus that interacts with Apc10 (Fig. 1 ▶ *d*). A rod-like density feature within the SC8-defined density that incorporates Apc2 closely matched the atomic model of Apc2. Docking Apc2 into this density positions the C-terminus of Apc2 adjacent to Apc10, which we previously docked using the subunit-deletion approach (Fig. 4 ▶; da Fonseca *et al.*, 2011 ▶; Schreiber *et al.*, 2011 ▶).

The third approach to assigning EM densities to subunits applies when there are no existing atomic models and the subunit-deletion approach is not possible. For example, there are no crystal structures or homology models for Apc4; however, having defined the position of all other large APC/C subunits we could define the electron density corresponding to Apc4 by a process of elimination. Apc4 is an 80 kDa protein; just a little too small to analyse by negative-stain electron microscopy, which is restricted to proteins larger than 100 kDa. To aid structural analysis of Apc4 by negative-stain electron microscopy, we generated monoclonal antibodies to Apc4, and the Fab–Apc4 complex is now of sufficient size to analyse by EM. Fig. 5 ▶(*a*) shows a gallery of two-dimensional class averages of the Apc4–Fab complex. The Fab is well resolved whereas Apc4 is less well defined overall, indicating that the Fab interacts with a flexible epitope on Apc4. Particular two-dimensional class averages show well defined two-dimensional projections in which structural details are clear. These averages resemble the electron density extracted from the holo APC/C cryo-EM map potentially corresponding to Apc4 (Fig. 5 ▶ *b*). The approach of generating Fab–protein complexes was adopted by Wu *et al.* (2012 ▶) to enable single-particle cryo-EM studies of small proteins (*i.e.* 100 kDa in size).

By combining these docking results, we generated a partial pseudo-atomic model of the APC/C. The model, based on the recombinant *S. pombe* APC/C cryo-EM structure, which is still being refined and is at about 12 Å resolution (Fig. 6 ▶). Here, we have docked the atomic models of APC/C subunits into the density and their positions have been refined using a rigid-body approach by means of *URO* (Navaza *et al.*, 2002 ▶; rigid-body fitting methods have been reviewed by Wriggers & Chacón, 2001 ▶). Flexible fitting approaches allow modelling of protein conformational changes and structural differences between the atomic model and the actual structure of the subunit within the context of the multi-subunit assembly (recently reviewed by Trabuco *et al.*, 2009 ▶). Such approaches have been implemented in *Situs* (Wriggers & Chacón, 2001 ▶), *MDFF* (which employs molecular-dynamics simu­lations; Trabuco *et al.*, 2008 ▶) and *DEN*, which uses a deformable elastic network model with restraints imposed by the EM map (Schröder *et al.*, 2007 ▶, 2010 ▶; Brunger *et al.*, 2012 ▶). DiMaio *et al.* (2009 ▶) reported the refinement of protein structures into low-resolution density maps using *Rosetta*, achieving near-atomic resolution starting with EM density maps at 4–6 Å resolution. Other flexible fitting methods include those reported by Topf *et al.* (2008 ▶) that employ Monte Carlo search, conjugate-gradient minimization and simulated-annealing molecular-dynam­ics approaches. A method for cross-validation in cryo-EM-based modelling has recently been proposed (Falkner & Schröder, 2013 ▶).

In the instance of the APC/C, over 80% of residues were fitted into the EM map. The coordinates fit well into the EM map. Fig. 6 ▶ shows that three canonical TPR subunits, Cdc27, Cdc16 and Cdc23, which are homodimers and structurally related, stack in parallel on one side of the complex. Together they generate a quasi-twofold-symmetrical structure. The N-­terminal cullin repeats of Apc2 fit into a long rod-like density placing the C-­terminal domain and associated RING subunit Apc11 in close proximity to the subunit-recognition module of Apc10 and coactivator. The subunits for which we have not fitted atomic models are Apc4, Apc5 and the N-­terminus of Apc1, and their densities are shown in blue, red and purple, respectively (Fig. 6 ▶).

In the future we need to complete the model by determining the crystal structures of Apc4 and the N-terminus of Apc1, and we are aiming to extend the resolution of the EM maps to at least allow the definition of secondary-structural elements. Ideally, we would like to achieve a near-atomic resolution of <3.5 Å. Although this has been achieved for viral structures with high symmetry (60-fold; Zhang, Jin *et al.*, 2010 ▶), it is not clear whether this is possible with asymmetric structures, although a recent 4.5 Å resolution structure of the *Escherichia coli* ribosome indicates that cryo-EM techniques provide the potential to determine near-atomic resolution structures of asymmetric particles (Bai *et al.*, 2012 ▶). This paper and other recent studies (Li *et al.*, 2013 ▶; Campbell *et al.*, 2012 ▶) take advantage of the high signal to noise achieved by direct electron detectors. Direct electron detectors also allow ‘movie’ processing of frames to correct for beam-induced particle motion and also ameliorate loss of resolution owing to radiation-induced sample damage. In addition to improvements in detectors, microscope technology and software [for example, *RELION*, which implements a Bayesian approach to cryo-EM reconstructions (Scheres, 2012*a* ▶,*b* ▶)], biochemical approaches to rigidify multi-subunit complexes will also be important.

As well as using atomic models mainly derived from crystallo­graphy to interpret EM-derived electron-density maps, it is also possible to use EM maps to obtain phase information for crystal structure determination. This approach was used by Steitz and coworkers to phase crystals of the large ribosomal subunit (Ban *et al.*, 1998 ▶). We tested whether we could use the Cdc16–Cdc26-assigned density of our EM map of *S. pombe* APC/C to phase Cdc16–Cdc26 crystals which we had previously determined using SAD methods (Zhang, Kulkarni *et al.*, 2010 ▶). Although there is little resolution overlap between the crystallographic data and the EM data since typical EM data sets range from 8 or 10 to 200 Å resolution, whereas crystallographic data range from 2–3 to 15–20 Å resolution, this approach worked well (Fig. 7 ▶ *a*). To test whether the Cdc16-assigned density could be used as a search model in molecular replacement, we placed the EM map corresponding to Cdc16–Cdc26 into a large *P*1 cell five times the map dimensions to calculate the structure factors (Fig. 7 ▶ *c*). The larger size of the cell was to ensure that cross-Patterson vectors calculated for the model, for this particular unit cell, are larger than the radius of integration. Placing the model in a large *P*1 cell would also improve the signal-to-noise ratio in molecular replacement. Before calculating structure factors, the EM map was not subject to scaling corrections. For molecular-replacement calculations, a resolution cutoff of 4–30 Å was applied to the diffraction data. Phases obtained from molecular replacement using *Phaser* (McCoy *et al.*, 2007 ▶) were extended from 10 to 5 Å resolution, allowing clear definition of α-helices in the crystal structure (Fig. 8 ▶). This result validated the quality of the EM-derived density maps. We are keen to apply this technique to provide phase information to determine the Apc4 crystal structure.

In summary, we can dock models into EM maps through a difference density approach. The advantage of this method is that is provides empirical constraints to docking and provides information on both the molecular boundaries and position of subunits. A limitation of this approach is that it is dependent on the ability to generate stable subcomplexes. The second approach is to dock by visual inspection, and a third approach, when atomic models are not available, is to use negative-stain EM of individual subunits, possibly aided by generation of subunit–Fab complexes. Generation of a pseudo-atomic model requires atomic models of individual subunits *via* crystallo­graphy or NMR or from structural homologues, and initial docking can be refined by rigid-body refinement and flexible fitting using programs such as *URO* (Navaza *et al.*, 2002 ▶) and *MDFF* (Trabuco *et al.*, 2008 ▶, 2009 ▶). Ultimately, if the resolution is sufficient, it is possible to determine structures *ab initio* using EM data.

## Figures and Tables

**Figure 1:**
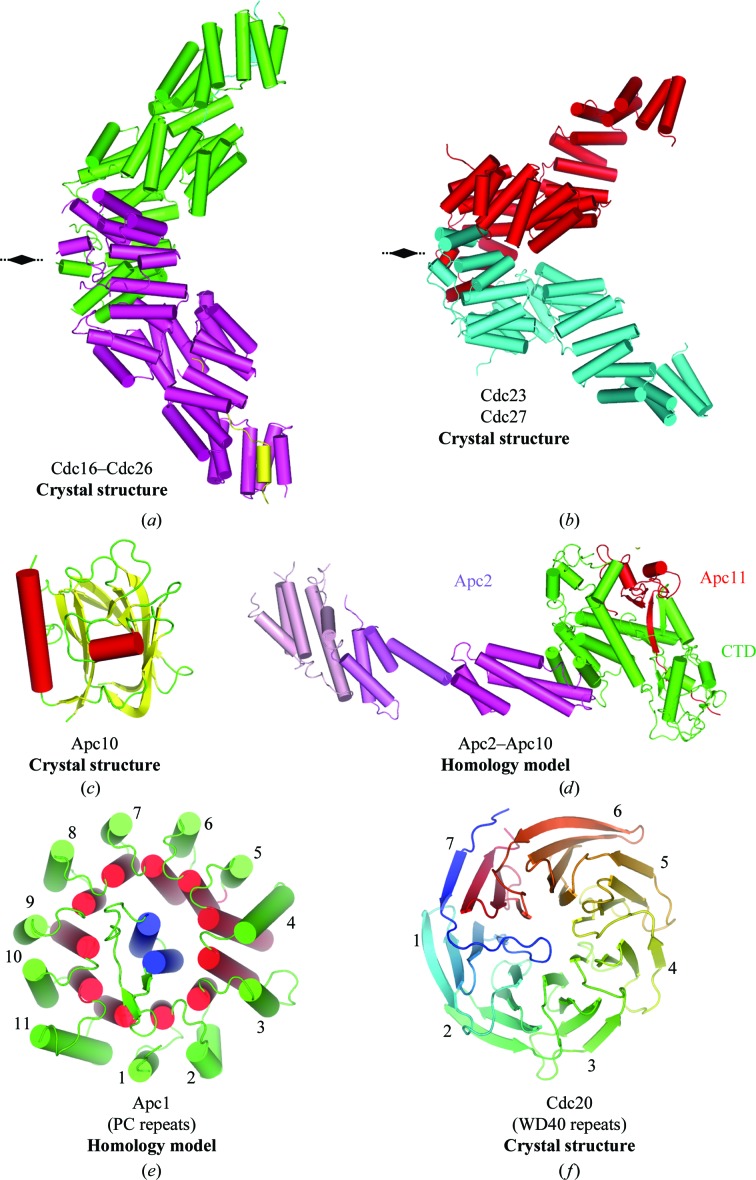
Gallery of APC/C subunit structures. (*a*) Cdc16–Cdc26 complex (PDB entry 2xpi; Zhang, Kulkarni *et al.*, 2010 ▶). (*b*) Cdc23 (PDB entry 3zn3; Zhang *et al.*, 2013 ▶) and Cdc27 (PDB entry 3kae; Zhang, Roe *et al.*, 2010 ▶) have similar structures to Cdc16. (*c*) Apc10 (PDB entry 1gqp; Au *et al.*, 2002 ▶). (*d*) Model of the Apc2–Apc11 complex modelled on Cul1–Rbx1 of the SCF complex (PDB entry 1ldk; Zheng *et al.*, 2002 ▶). (*e*) PC domain of Apc1 modelled on Rpn2 (PDB entry 4ady; He *et al.*, 2012 ▶). (*f*) WD40 domain of Cdc20 (PDB entry 4aez; Chao *et al.*, 2012 ▶).

**Figure 2:**
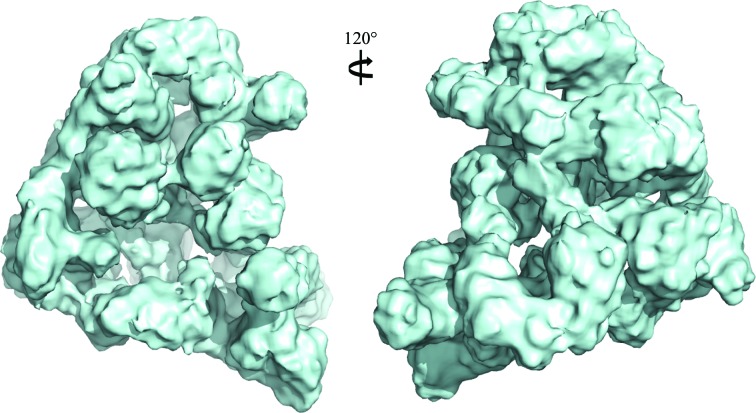
Two views of the cryo-EM reconstruction of *S. cerevisiae* APC/C in complex with coactivator Cdh1 and a high-affinity D-box peptide (da Fonseca *et al.*, 2011 ▶).

**Figure 3:**
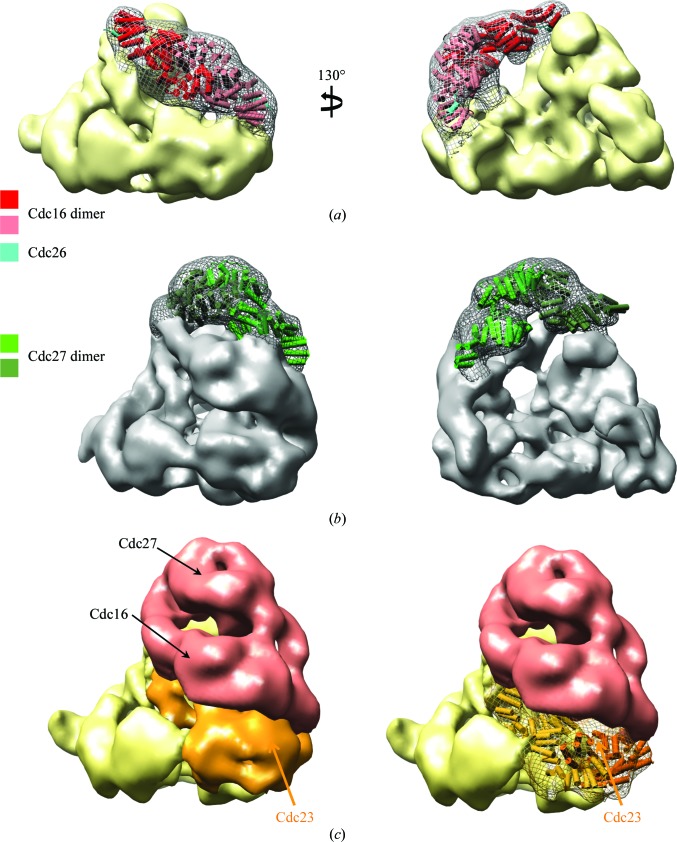
Examples of the subunit-deletion approach to define the location and structure of APC/C subunits. (*a*) Two views showing the Cdc16 difference density in mesh with Cdc16 coordinates superimposed. (*b*) Two views showing the Cdc27 difference density in mesh with Cdc27 coordinates superimposed. (*c*) Overlapping densities from two APC/C subcomplexes that share Cdc23 in common defines Cdc23 (orange). Cdc23 coordinates are shown in the right panel (Schreiber *et al.*, 2011 ▶). TPR6 corresponds to the red plus orange volume, whereas SC8 corresponds to the yellow plus orange volume.

**Figure 4:**
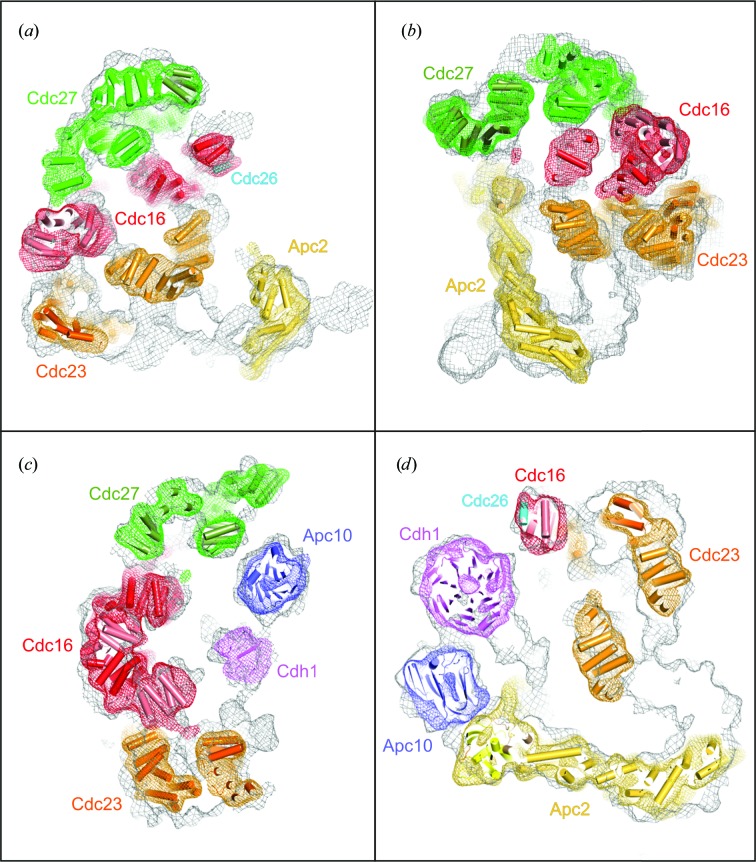
Identification of APC/C subunits by visual inspection. In (*a*), (*b*) and (*d*) rod-like density defines Apc2 (da Fonseca *et al.*, 2011 ▶).

**Figure 5:**
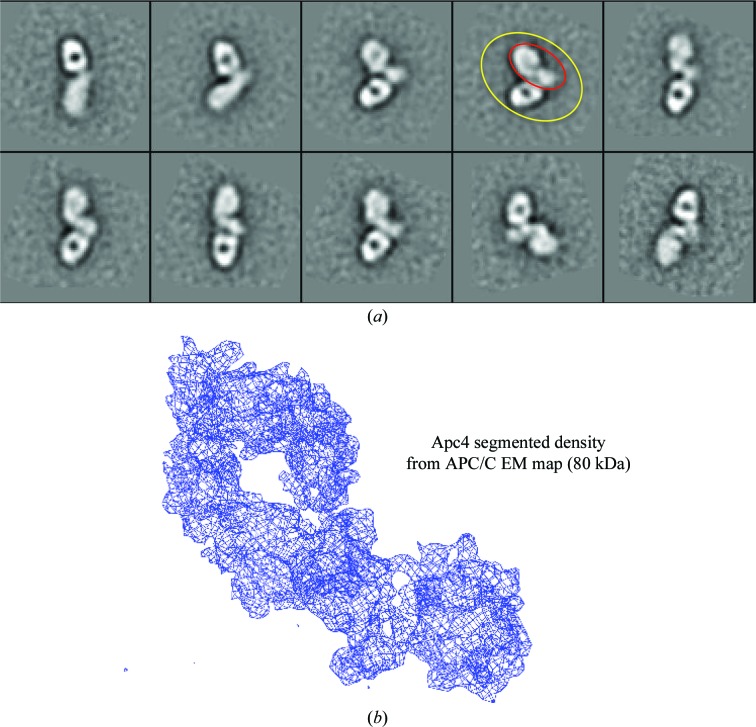
Apc4 definition. (*a*) Representative two-dimensional class averages from negative-stain EM micrographs of an Apc4–Fab complex. In one representative class, the Apc4–Fab complex is circled in yellow. Apc4 is circled in red. (*b*) Density map corresponding to Apc4 and extracted from *S. pombe* APC/C.

**Figure 6:**
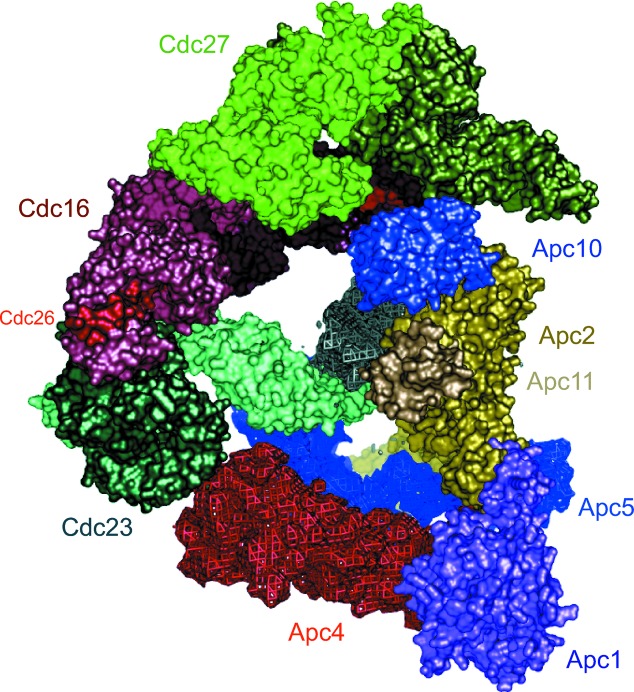
Pseudo-atomic model of the *S. pombe* APC/C. Fitted subunits (Apc2, Apc11, Apc10, Cdc16, Cdc23, Cdc27 and Cdc26) are shown with molecular surfaces. Density assigned to Apc1, Apc4 and Apc5 is shown.

**Figure 7:**
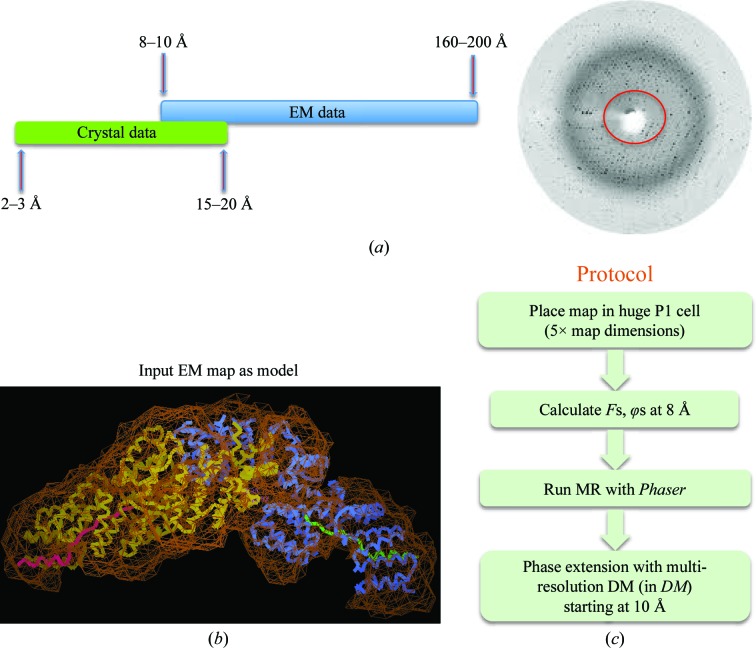
MR structure solution with an EM map as a model. (*a*) Schematic of the overlap of EM and X-ray data. (*b*) Cdc16–Cdc26 assigned density with coordinates. (*c*) Protocol for using an EM map for crystallographic molecular replacement.

**Figure 8:**
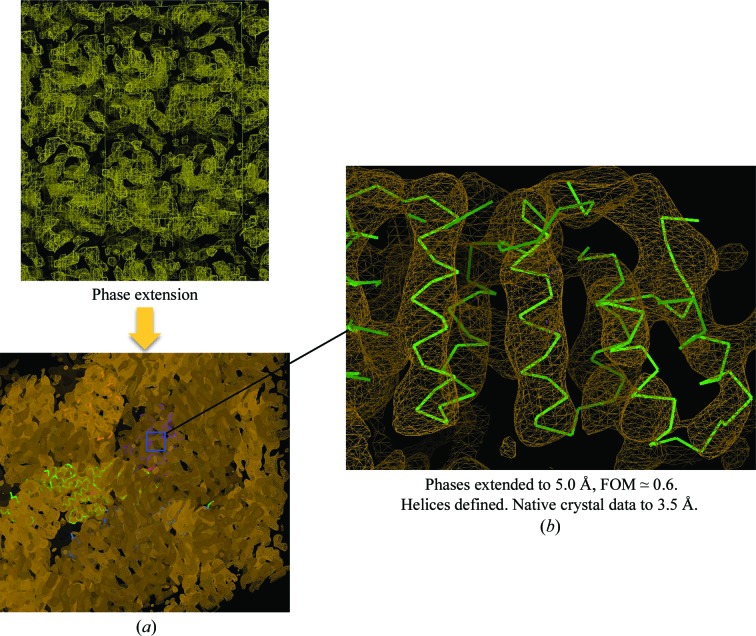
Cdc16–Cdc26 structure solution with an EM map as a model. (*a*) The initial map calculated to 10 Å resolution was extended to 5 Å. (*b*) The final map shows clear indications of α-helices.

**Table 1:** Subunits of the APC/C ND, not defined.

*Homo sapiens*	Molecular mass (kDa)	*N*	*S. cerevisiae*	*S. pombe*	Structural motif	Function	Model determination	Mapping approach
Core subunits								
Apc1	216.5	1	Apc1	Cut4	α-Helices and PC repeats	Scaffolding subunit	PC domain homology	Docking/elimination
Apc2	93.8	1	Apc2	Apc2	Cullin homology	Catalytic/Apc11 binding	Homology	Docking
Apc3	91.9	2	Cdc27	Nuc2	TPR	Cdh1/Apc10 binding	X-ray/homology	Subunit deletion
Apc4	92.1	1	Apc4	Lid1	Unknown	Scaffolding subunit	ND	Fab-Apc4 EM negative stain
Apc5	85.1	1	Apc5	Apc5	Extended TPR	Scaffolding subunit	Homology	Elimination
Apc6	71.7	2	Cdc16	Cut9	TPR	Scaffolding subunit	X-ray	Subunit deletion
Apc7	66.9	2	—	—	TPR	Cdh1/Apc10 binding	X-ray/homology	Subunit deletion
Apc8	68.8	2	Cdc23	Cut23	TPR	Scaffolding subunit	X-ray/homology	Subunit deletion
—	—	—	Apc9	—	Unknown	Cdc27 stabilizing		
Apc10	21.2	1	Apc10/Doc1	Apc10	Doc homology/IR motif	Substrate recognition	X-ray	Subunit deletion
Apc11	9.8	1	Apc11	Apc11	RING H2	Catalytic/E2 binding	Homology	ND
Apc13	8.5	1	Apc13/Swm1	Apc13	Unknown	Cdc23 stabilizing	ND	ND
—	—	—	—	Apc14	Unknown	Unknown		
Apc15	14.3	1	Mnd2	Apc15	Unknown	MCC/Ama1 regulator	ND	ND
Apc16	11.7	1–2	—	—	Unknown	TPR stabilizing	ND	ND
Cdc26	9.8	2	Cdc26	Hcn1	Extended chain/α-helix	Cdc16 stabilizing	X-ray	Subunit deletion
Coactivators								
Cdc20	54.7	1	Cdc20	Slp1	WD40/IR motif	Substrate recognition		Subunit deletion
Cdh1	55.2	1	Cdh1	Ste9	WD40/IR motif	Substrate recognition		Subunit deletion
—	—	—	Ama1	Mfr1	WD40/IR motif	Substrate recognition		ND
